# Effect Mechanism of Electroacupuncture at ST36 on the Injured Extensor Digitorum Longus in the Jumping Rat Model Based on mRNA-Seq Analysis

**DOI:** 10.3390/biomedicines9121849

**Published:** 2021-12-07

**Authors:** Qianqian Liu, Yanwei Guo, Rui Han

**Affiliations:** 1 School of Physical Education and Sport, Henan University, Kaifeng 475001, China; liuqianqian@henu.edu.cn; 2 Sports Reform and Development Research Center, Henan University, Kaifeng 475001, China; 3 School of Computer and Information Engineering, Henan University, Kaifeng 475001, China; guoyanwei@henu.edu.cn

**Keywords:** inflammation, transcriptomic, electroacupuncture stimulation, longitudinal jump training, cytokine-inducible SH2 containing protein (CISH)

## Abstract

The key target and regulatory mechanism of electroacupuncture of Zusanli (ST36) on extensor longus muscle injury in a jumping rat model were investigated. To this end, 24 female SD rats were randomly divided into the following four groups: no-treatment control group (NON), 6-week jumping group (J6O), electroacupuncture group after 6-week jumping (J6A), and natural recovery group after 6-week jumping (J6N). After 6 weeks of jumping, in the electroacupuncture group (J6A), electroacupuncture stimulation was applied at Zusanli(ST36) for 20 min per day over the course of 5 days. In the natural recovery group (J6N), rats were fastened with a special apparatus without electroacupuncture stimulation for 20 min at the same time. Transmission electron microscopy, transcriptome sequencing and analysis, Western blotting assay and immunofluorescence staining were performed at the end of our experiment. The recovery effect of J6A rats was more obvious than that of J6N rats and J6O rats as indicated by changes of infiltration of inflammatory cells and morphological structure. Notably, the morphological structure of J6A rats was closer to NON rats in the observation of transmission electron microscopy. CISH/STAT3 regulation was identified by mRNA-seq. The pro-inflammatory response to STAT3 activation was alleviated through up-regulating the expression of CISH protein in J6A rats relative to J6O rats. The level of BAX was decreased and the level of Bcl-2 level was increased in J6A rats relative to J6O rats. Moreover, when compared to J6N rats, the level of Bcl-2 was significantly up-regulated in J6A rats. Increased caspase-3 expression but decreased CDKN2α expression was shown in J6A rats relative to NON rats. These results indicate that the potential mechanism underlying electroacupuncture stimulation of Zusanli (ST36) in repairing the injured extensor digitorum longus following overused jumping may be attributed to CISH/STAT3 regulation of proteins associated with inflammation, apoptosis, and proliferation.

## 1. Introduction

Muscle fatigue due to reduced muscle shock absorption is the main cause of sports injuries. Overuse of the lower extremities resulted in lower leg injury rates of 9.0% to 32.2%, and the incidence of injuries around the foot ranged from 5.7 to 39.3% [1]. Muscle fatigue combined with repetitive motion loads during long-distance running and repetitive jumping movements can lead to fatigue of the dorsiflexors and plantar flexors of the ankle joint. The Mizrahi study [2] found that the mean integrated electromyographic signal (iEMG) of the anterior tibia was significantly lower from the 20th minute compared to the 5th minute of running, while no significant changes were found in the iEMG of the gastrocnemius muscle, suggesting that the imbalance between muscles may increase the risk of skeletal stress fractures. It has been reported that the change in toe orientation during vertical jumping significantly affects the knee biomechanics in the anterior and horizontal plane [3]. Existing evidence also supports that knee abduction moments, angles and internal tibial rotation angles during landing are risk factors for anterior cruciate ligament injury of the knee.

The extensor digitorum longus plays an important role in the dorsiflexion of the foot and extension of the big toe, determining the range of motion and intensity of the ankle and big toe [4]. Muscle fatigue or injury of the extensor digitorum longus can reduce calf and ankle exercise capacity, causing sports injuries to the foot, ankle, or knee. An increasing number of studies have shown that [5,6,7] increased lower extremity stiffness is associated with improved athletic performance, such as jumping, throwing, endurance running, sprinting, and changing direction. Lower extremity stiffness requires a complex interaction of muscles, tendons, ligaments, cartilage, and bone. Most tissues return to their original state when the force that triggers the deformation decreases or stops, and these tissues are known as “elastic tissues” including tendons and muscles [8]. Tendons have a high energy return rate and efficiency during recoil between 65 and 90% [9] whereas there the return rate of muscle is approximately 60% in muscle [10]. It is more important to train the muscles than the tendons to obtain the best elastic energy return during exercise, because when two springs of different stiffness are placed in series, more energy is stored in the supple spring [8]. Therefore, the maintenance of muscle structure and function is very important for athletic performance and health. In this study, we used an electrical stimulation jumping device to make rats perform repeated jumping exercises to result in the injury of the extensor digitorum longus. Then we investigated repair mechanism of the injured extensor digitorum in response to electroacupuncture at Zusanli (ST36) based on mRNA-seq analysis.

## 2. Materials and Methods

### 2.1. Animals and Grouping

This animal experimental content and exercise protocol were approved by the Ethics Committee of Henan University (approval number: HUSOM2021-174, approval date: 15 April 2021). In total, 24 female adult Sprague Dawley rats aged 12 weeks (weight 280 ± 22 g), were purchased from Henan Provincial Experimental Animal Center (License No. SCXK (Henan) 2017–0001). The mice were kept free to drink water during the experiment and the bedding was changed 2–3 times a week. Ambient temperature is 20 °C–22 °C, no humidity control, and natural light. The experimental animals were randomly divided into a control group (NON), a six-week jumping group (J6O), a six-week jumping + electro-acupuncture group (J6A), and a six-week jumping + natural recovery group (J6N), with six animals in each group.

### 2.2. Animal Training and Acupuncture Methods

The rat-conditioned reflex chamber was customized with reference to the literature [11], and included a reflex box (with a copper strip stimulation plate, a catch dirt box, a stimulation shuttle isolation channel, and an experimental platform), a stimulation regulator manipulator, and a wireless rocking control (control range, 0–20 m). The stimulation intensities were as follows: 0–120 V, 220 V, and 50 Hz AC. Regarding the procedure, the rat was placed on the copper bar stimulation plate in the reflex box. The power connection button of zone I and (or) zone II of the shaker was pressed, and the stimulation intensity was between 20 and 50 V. The electrical stimulation triggered the rat to jump to the upper safety platform until it jumped to the safety platform to avoid the electrical stimulation, and then put the rat back to the original position for the next jump after jumping to the safety platform to avoid electrical stimulation. After jumping to the safety platform, the rat was then returned to the original position for the next jump. The height of the platform was used to monitor the jumping height of the rats; successfully jumping to the platform to avoid electrical stimulation was considered as one jump, with a platform height of 20 cm. Acclimatization training was performed before the formal experiment, and the number of jumps was incremented during the week of acclimatization training. During formal training, the rats performed 150 jumps per day; the rats were divided into 15 groups, with 10 jumps per group, and 2 min rest between groups. Training was conducted 5 days per week with 2 days of rest for 6 weeks. After 6 weeks of jump training, the J6O group was rested for 12 h before the samples were taken. At the same time the J6A group was subjected to electroacupuncture of the Zusanli (ST36), perpendicular insertion 7 mm, the needle handle was connected to the electroacupuncture instrument, the intensity was measured by the slight trembling of the rat’s limbs (1–2 mA), sparse and dense waves of 2 Hz/15 Hz, and an output voltage of 2 V. Electroacupuncture stimulation was performed for 20 min per day for 5 days. The J6N group was fixed for 20 min at the same time every day, without electroacupuncture. After electroacupuncture, the rats were allowed to rest for 12 h, and the two groups were sampled in parallel.

### 2.3. Sampling

Experimental animals were executed by over-anesthesia following completion of the corresponding interventions in each group. The rats’ skin was circumscribed along the ankle of the hind limb to expose the calf muscles, and the extensor digitorum longus was separated and clipped from the upper and lower tendons. Some of the extensor digitorum longus was rapidly cut into 1 mm^3^ cubes, and placed in glutaraldehyde to prepare electron microscopic sections.

The left extensor digitorum longus was fixed in 4% paraformaldehyde for 48 h before immunofluorescence staining. The right extensor digitorum longus was labeled and snap frozen in liquid nitrogen for Western blotting.

### 2.4. Histomorphological Observation

The extensor digitorum longus was routinely sectioned and morphologically observed by transmission electron microscope and immunofluorescence staining. Antibodies information is as follows: BAX (BM3961, BOSTER), BCL-2 (GB 12008 Servicebio).

### 2.5. Transcriptomic Analysis

**Figure 1:**
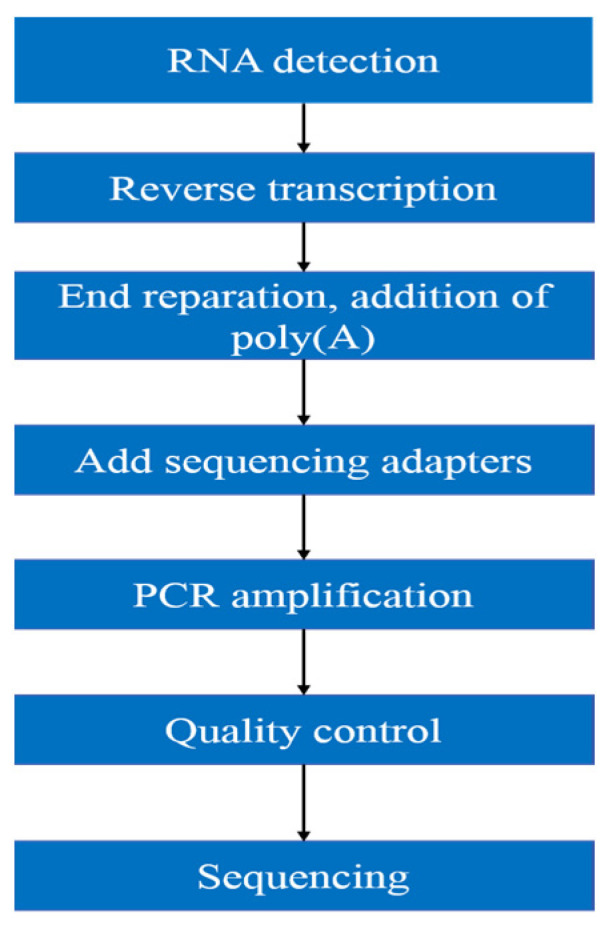
mRNA sequencing workflow.

As shown in the following Figure 1, the workflow of mRNA sequencing includes sample preparation, library construction, library quality control, and sequencing.

Purity, concentration and integrity of RNA sample were examined by NanoDrop, Qubit 2.0, Agilent 2100, etc. Only RNA with good quality could move on to following procedures. (1) mRNA was isolated by Oligo(dT)-attached magnetic beads. (2) mRNA was then randomly fragmented in fragmentation buffer. (3) First-strand cDNA was synthesized with fragmented mRNA as template and random hexamers as primers, followed by second-strand synthesis with addition of PCR buffer, dNTPs, RNase H, and DNA polymerase I. Purification of cDNA was processed with AMPure XP beads. (4) Double-strand cDNA was subjected to end repair. Adenosine was added to the end and ligated to adapters. AMPure XP beads were applied here to select fragments within size range of 300–400 bp. (5) cDNA library was obtained by certain rounds of PCR on cDNA fragments generated from step 4. In order to ensure the quality of library, Qubit 2.0 and Agilent 2100 were used to examine the concentration of cDNA and insert size. Q-PCR was processed to obtain a more accurate library concentration. Library with concentration larger than 2 nM is acceptable. The qualified library was pooled based on pre-designed target data volume and then sequenced on Illumina sequencing platform.

### 2.6. Western Blotting

Each rat extensor digitorum longus was taken for the extraction and quantification of cytoplasmic proteins, and the protein samples of each group were subjected to constant pressure at 4% gel concentrate 80 V for 30 min, followed by a constant voltage of 150 V for 70 min and a constant current of 300 mA for 120 min at 12–15% separation gel. 5% skimmed milk powder was closed at 4 °C for 6 h and primary antibody was incubated overnight; then transferred to PVDF membrane. Then incubate the secondary antibody at 1:1000 dilution for 90 min at room temperature. The membranes were rinsed three times with TBST for 10 min each, and finally the membranes were rinsed two times with TBS for 10 min each. The target proteins were detected using ECL luminescence. The corresponding grayscale values were read, with GAPDH as an internal reference for cytoplasmic proteins, and the relative grayscale values of the target proteins were calculated. Details of the antibodies used are provided below (Table 1).

### 2.7. Statistical Analysis

Data were expressed as mean (±SD), and data were analyzed by two-way analysis of variance. The two factors were the treatment factor (normal control or jump training) and the intervention factor (6-week injury natural recovery; 6-week injury electroacupuncture stimulation), *p* < 0.05 and *p* < 0.01 were considered significant difference.

## 3. Results

### 3.1. Ultrastructural Analysis

**Figure 2:**
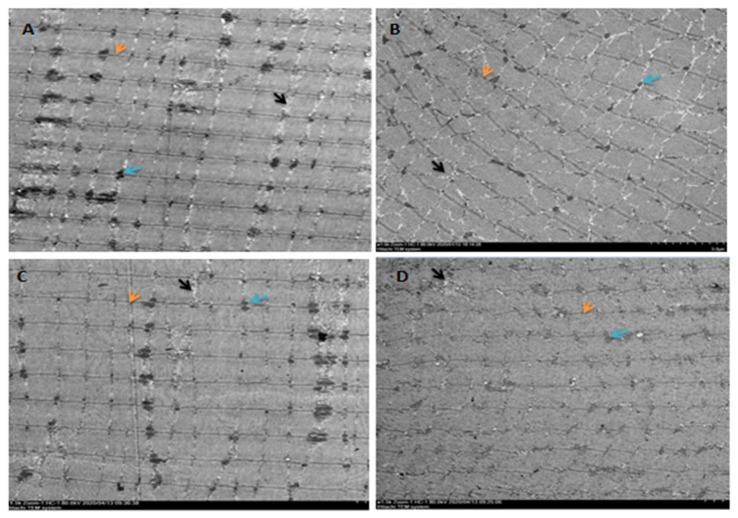
Extensor digitorum longus observation by electron microscope. (**A**): control group (NON); (**B**): six-week jumping group (J6O); (**C**): six-week jumping + electro-acupuncture group (J6A); (**D**): six-week jumping + natural recovery group (J6N). All images were obtained at the same original direct magnification (×1500). Notes: orange arrow: Z line; blue arrow: mitochondria; black arrow: collagen fibers.

Electron microscopic observation of the extensor digitorum longus in NON rats showed normal membrane and myofibrillar architecture, including neatly aligned muscle fibers, intact Z-lines, an appropriate number of extracellular matrix collagen fibers evenly distributed around the myocytes, and small intercellular collagen area (Figure 2A). J6O rats showed obvious signs of damaged or disordered myofibrillar architecture, such as disorganized myogenic fibers, swelling and rupture of mitochondria, abnormal mitochondrial structure, increased collagen area, and interstitial edema (Figure 2B). After 5 days of electroacupuncture stimulation, the myogenic fiber morphology in the J6A rats recovered significantly, as shown in Figure 2C, with normal arrangement of myofibrils, intact Z-bands, decreased collagen fibers, and an increased number of mitochondria with a normal structure. The structure of the myofibrils in the extensor digitorum longus of J6N rats was also repaired. However, compared to J6A rats, J6N rats had worse recovery, given that the Z-line was not aligned, the number of collagen fibers was less, the arrangement of collagen fibers was less neat, and the repair of mitochondrial structure was slight (Figure 2D).

### 3.2. Transcriptome Data Processing and Quality Control Analysis

After transcriptome sequencing of 12 samples, clean data were obtained by filtering the dismount data. A total of 86.85 GB clean data were obtained; the clean data of all samples comprised up to 6.03 GB, and the percentage of Q30 bases was ≥94.17%. Certain amount of filtered data were randomly compared with the NT library, and no contamination was detected. Next, the clean reads of each sample were compared with the assigned reference genome, and the comparison efficiency ranged from 94.84% to 95.69%. Then, the gene expression was analyzed on the basis of the comparison results. Next, the differentially expressed genes were identified according to the number of genes expressed in different samples, and functional annotation and enrichment analyses were performed (Table 2).

### 3.3. Screening Genes and Pathways through GO/KEGG

**Figure 3:**
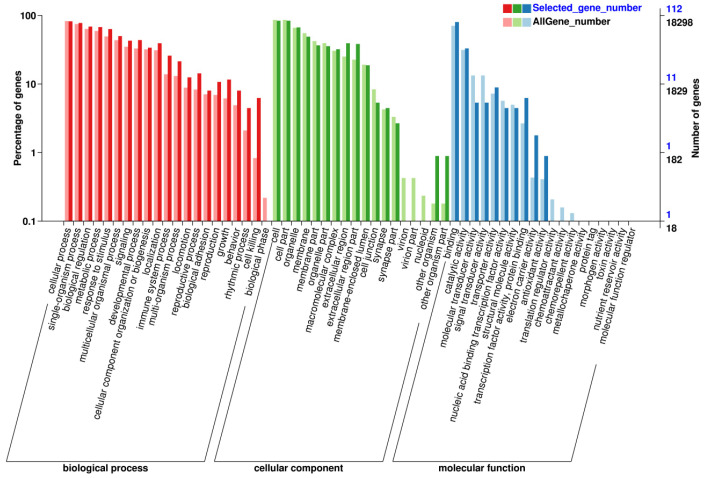
GO functional enrichment analysis of differential genes.

After screening for differential genes of extensor digitorum longus muscle from each sample, GO function enrichment analysis was performed, and a total of 58 entries were obtained, including 21 for Biological Process analysis, 18 for Cellular Component analysis, and 19 for Molecular Function analysis. The differential genes for each entry are shown in Figure 3. The horizontal coordinates represent specific entries; the vertical coordinates represent the number of genes enriched in this entry, and the top entries are displayed in a bar graph. These entries in the GO function enrichment analysis were related to (1) BP analysis: Biological process; (2) CC analysis: Cellular component; (3) MF analysis: Molecular function. It is suggested that electroacupuncture stimulation deals with muscle damage mainly through the highly expressed biological processes to promote the repair in rats. 

**Figure 4:**
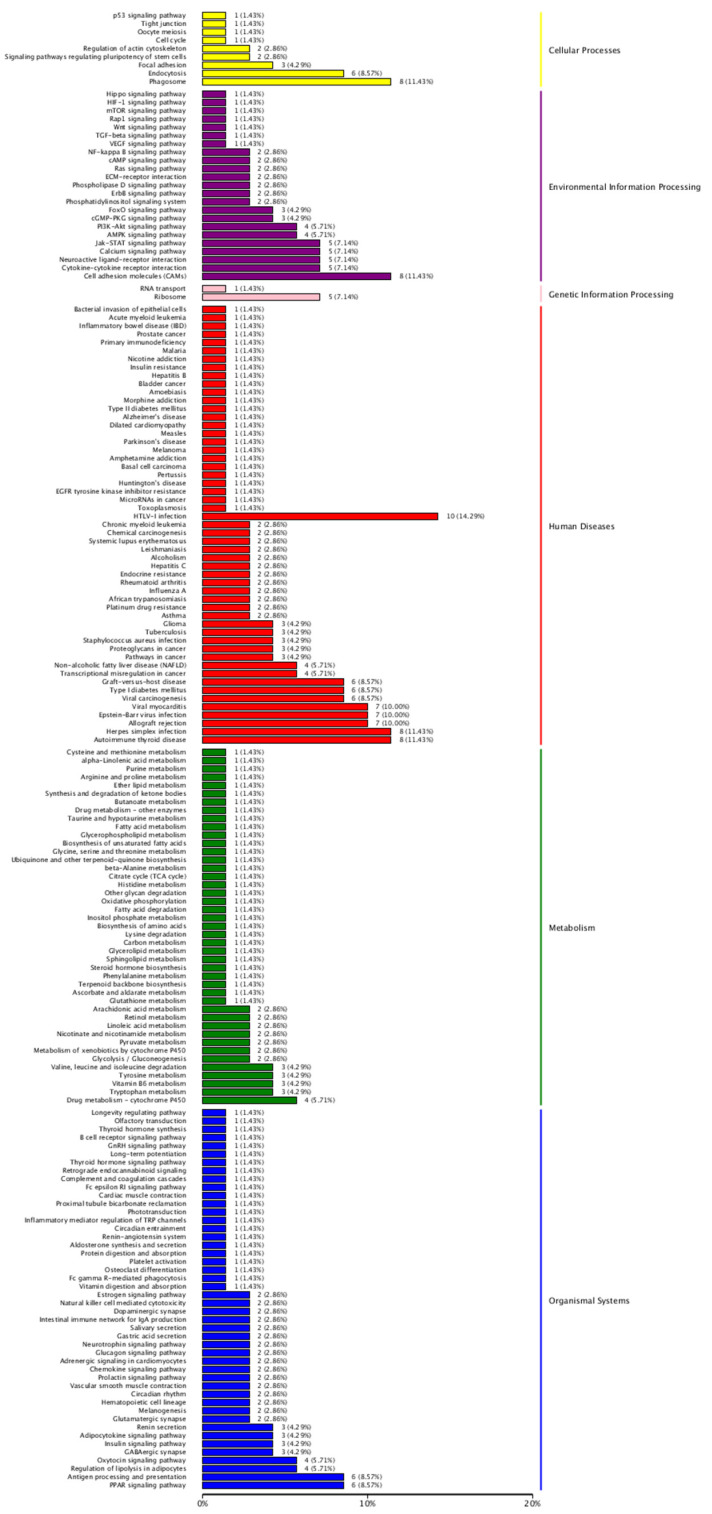
KEGG functional enrichment analysis of differential genes.

Similarly, KEGG pathway enrichment analysis was performed and 177 pathways were obtained, including 9 for Cellular Processes analysis, 22 for Environmental Information Processing analysis, 2 for Genetic Information Processing analysis, 54 for Human Diseases, 44 for Metabolism, and 46 for Organismal Systems. The differentially expressed genes (DEGs) of each entry are shown in Figure 4, where the vertical coordinate represents the specific entry; the horizontal coordinate represents the number of genes enriched in this entry, where the length of the bar represents the number of genes, and the greater number of genes, the longer the entry.

### 3.4. Screening Major Target Pathways and Key Target Genes

**Figure 5:**
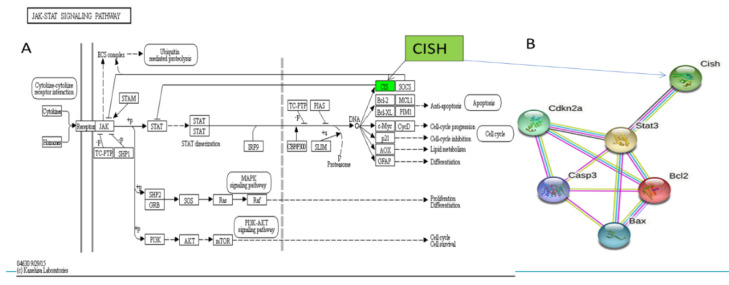
(**A**) Schematic diagram of targeted CISH gene in the JAK-STAT signaling pathway for improving muscle injury by electroacupuncture stimulation of Zusanli (ST36). (**B**) Interaction network prediction of multiple proteins. Notes: The gene shown in green text box is up-regulated in (**A**).

The highly expressed signaling pathways of KEGG were compared by searching them in TOP GO, showing that differentially expressed genes were significantly enriched in the Janus kinase-signal transducer and activator of transcription signaling (JAK/STAT) pathway (Table 3). The JAK/STAT signaling could be negatively regulated by Suppressors of Cytokine Signaling (SOCS) family through competing binding to JAKs to inhibit kinase activities, covering STAT binding sites of cytokine receptors, or degrading targeted proteins (Table 3). SOCS proteins contain 8 members involving cytokine-inducible SH2 containing protein (CISH) and SOCS1–7. Then, CISH gene was identified from DEGs in our study (Figure 5A). Figure 5A shows CISH expression was up-regulated by electroacupuncture stimulation, and Figure 5B shows the protein interactions of CISH, Cas-3, STAT3,CDKN2α,BCL-2 and BAX, indicating that CISH acts as an upstream control gene in STAT3 in the JAK/STAT signaling pathway to regulate proliferation, apoptosis, and anti-apoptosis in response to muscle injury repair.

### 3.5. Changes in Key Target Proteins Expression from Each Group

**Figure 6:**
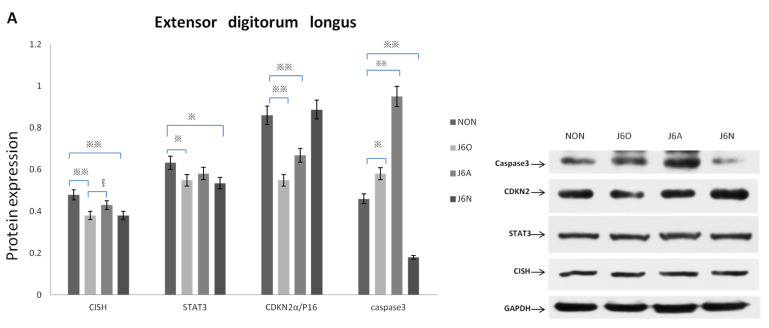

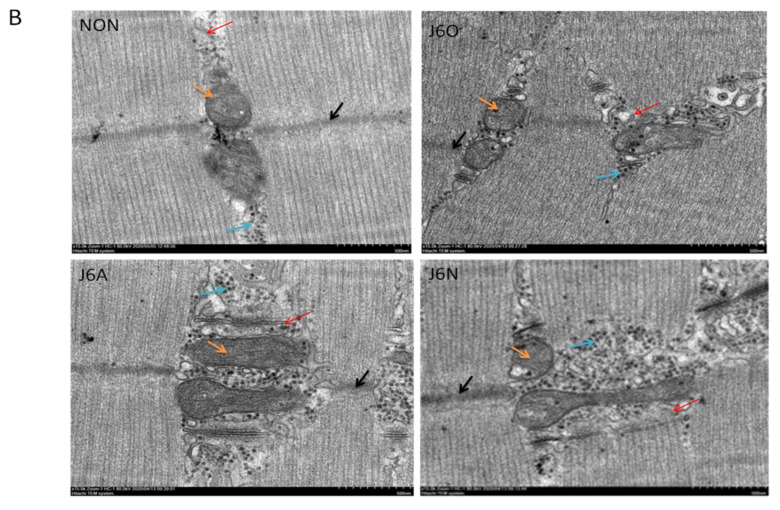
Expression of key target proteins in the extensor digitorum longus (**A**) and the ultrastructural changes of the extensor digitorum longus (**B**) Notes: All images were obtained at the same original direct magnification (×15,000) ^※※^ and ^※^ respectively represent *p* ˂ 0.01 and *p* ˂ 0.05 compared to the NON group and § means *p* ˂ 0.05 compared to the J6O group. Black arrow: Z line. Orange arrow: Mitochondria, blue arrow: inflammatory factor. Red arrow: triad junction.

Figure 6A shows expressions of CISH, STAT3, CDKN2α, and caspase-3 in the extensor digitorum longus muscles from four groups by Western blotting assay.

When compared to NON rats, J6O rats had decreased expressions of CISH, STAT3, and CDKN2α and increased expression of Caspase-3 (*p* < 0.01, *p* < 0.05, *p* < 0.01, and *p* < 0.05, respectively). When compared to NON rats, J6A rats had a slight decline of CISH and STAT3 but with no differences. J6A rats had the decreased CDKN2α expression and increased Caspase-3 expression relative to NON rats (*p* < 0.01, *p* < 0.01, respectively). Moreover, also noteworthy was higher level of CISH in the J6A group as compared to the J6O group (*p* < 0.05). Expressions of CISH, caspase-3, and STAT3 were down-regulated in J6N rats relative to NON rats (*p* < 0.01, *p* < 0.05, and *p* < 0.01 respectively), while the expression of CDKN2α was slightly up-regulated with no difference.

We also observed the ultrastructure of the extensor digitorum longus by transmission electron microscopy with particular attention to the inflammatory response, transverse tubules, and mitochondrial changes (Figure 6B). Mitochondrial stress induced by repeated jumping movements can lead to the release of damage-associated molecular that activates innate immunity in rats. In the J6O group, disorganized muscle segments and reduced internal cristae density were displayed, indicating mitochondrial matrix swelling, multilayer structure formed by sarcoplasmic reticulum (SR), and the onset of inflammatory responses. Five days after training, when compared to the NON group, large mitochondria were beyond flanking the Z-band where the mitochondria are normally located and even spanned the entire myotome in the J6N group, also with formation of similar membrane stack-like structures by the SR. Simultaneously, more inflammatory cells were observed near the mitochondria in J6N rats. However, for J6A rats, the arrangement of the myofilaments restored to be neatly and structure of mitochondria and triads converged toward normalization, suggesting that the inflammatory response resulting from overused jumping training significantly diminished in the J6A group. 

### 3.6. Immunofluorescence Detection of Changes in BCL-2/BAX Expression in Each Group

**Figure 7:**
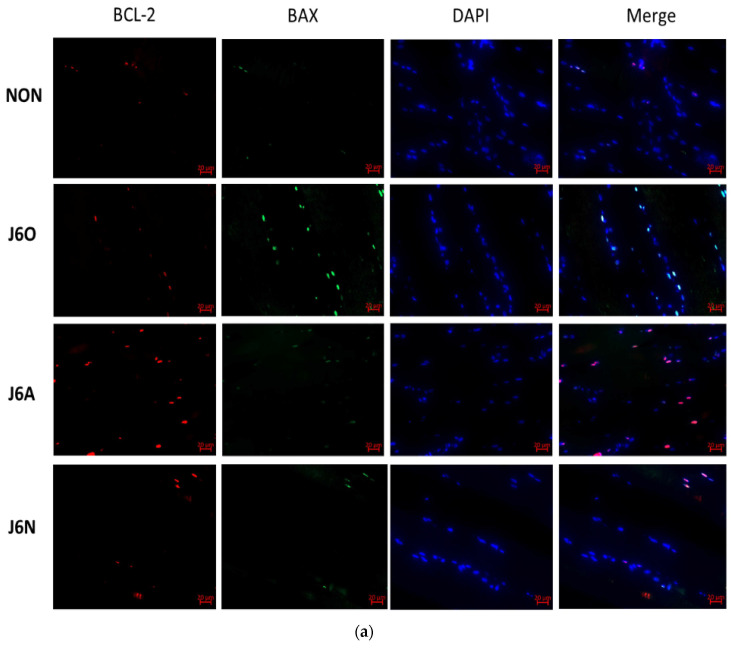

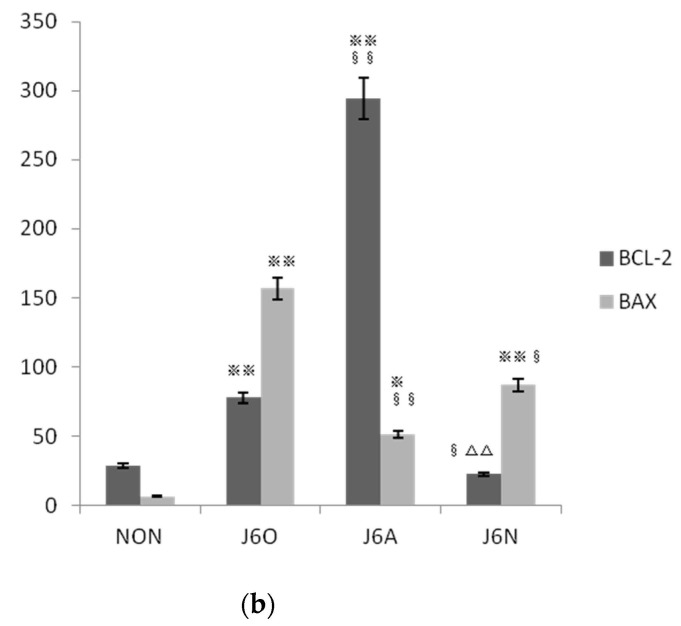
(**a**) Immunofluorescence detection of changes in the expression of BCL-2/BAX in extensor digitorum longus muscles of rats in each group and (**b**)Statistical analysis of immunofluorescence in each group (vs. NON, ^※※^ *p* ˂ 0.01, ^※^ *p* ˂ 0.05; vs. J6O, ^§§^ *p* ˂ 0.01, ^§^ *p* ˂ 0.05; vs. J6A, ^△△^ *p* ˂ 0.01).

Immunofluorescence staining was used for identifying the activation of Bcl-2 and BAX. As shown by Figure 7, expressions of BCL-2 and BAX in J6O and J6A groups significantly increased, and all of these were statistically significant as compared to the NON group (*p* < 0.01 or *p* < 0.05). Moreover, also noteworthy was higher level of BCL-2 (*p* < 0.01) and lower level of BAX (*p* < 0.01) in the J6A group as compared to the J6O group. Moreover, as compared to NON group, BCL-2 slightly decreased with no difference and BAX increased in the J6N group (*p* < 0.01). However, when compared to the J6A group, the level of BCL-2 significantly down-regulated (*p* < 0.01) and the level of BAX slightly up-regulated with no difference in the J6N group.

## 4. Discussion

### 4.1. Effects of Electroacupuncture at ST36 on the Morphological Structure of an Overused Extensor Digitorum Longus

Pain from overuse injuries or other causes is a major health problem, which has serious social and economic implications, with the potential to result in hundreds of billions of dollars in physician visits, pain medications, and loss of productivity each year. Conventional medical treatments may be only moderately effective and often have side effects. Indeed, opioids are common analgesic medications but often lead to problematic effects such as addiction, tolerance, nociceptive hypersensitivity, and end-organ damage. Hence, combined or alternative analgesic strategies including acupuncture are often used to alleviate pain after tissue damage. Acupuncture has been used in China for thousands of years, and the clinical effect of acupuncture or electroacupuncture stimulation on the inhibition of persistent pain such as inflammatory pain, neuropathic pain, visceral pain, and cancer-related pain has been supported by relevant scientific evidence [12,13,14,15,16]. In addition to pain-relief, both human and animal studies have confirmed the definite effect of electroacupuncture on movement disorders, and tissue regeneration, among others [17,18,19]. Similarly, we have found electroacupuncture stimulation can promote the healing process of damaged tissues. 

As shown by electron microscopy of J6O rats following excessive jump training, torn myofibers, disorganized structure of myoblasts, malalignment of myofibers, swollen and ruptured mitochondria, abnormal increase in the number and volume of collagen fibers, and interstitial edema were displayed. For 5 days with electroacupuncture stimulation, the ultrastructural morphology of J6A rats was closer to NON rats, as observed by neat arrangement of myofibers, intact Z-lines, and extracellular matrix collagen fibers evenly distributing around myocytes. The maintenance of native triads structure serves as a crucial role in skeletal muscle performance, as the triads of skeletal muscle are the basis of excitation-contraction coupling, and the transverse tubules are the extension of the body fascia. In the present study, it was found that repeated jumping caused swelling, disorder, vacuolation, and even fragmentation of the triads. After five days, electroacupuncture intervention promoted the conformation recovery of triads, the effect of which was better than that of the J6N group. It is suggested that electroacupuncture stimulation indeed plays an important role in restoring the structure of damaged triads [20]. Moreover, when compared to J6N rats, the number of collagen fibers was significantly reduced and the number of mitochondria was significantly increased with restored structure. These results indicate that electroacupuncture stimulation indeed promotes structural recovery of the injured extensor digitorum longus arising from excessive jumps (Figure 2).

### 4.2. Molecular Mechanism of Electroacupuncture Stimulation on the Repair of Damaged Muscle in Overused Jumping Rats 

One research has provided the evidence that electroacupuncture makes the gene expression normalized in patients’ skeletal muscle [14]. To uncover the potential molecular mechanism of electroacupuncture on overused muscle, we performed transcriptomic analyses and targeted the cytokine-inducible Src homology 2-containing protein (CISH) from differentially expressed genes and the Janus kinase-signal transducer and activator of transcription signaling (JAK/STAT) pathway through KEGG and Top Go analysis. 

CISH is a negative feedback regulator of signaling pathways induced by specific cytokines and growth factors. It was reported many years ago that CISH disrupted the JAK/STAT5 activity inducing by IL-2 through the combination of IL-2β [21], and it has since been reported that CISH can regulate the activity of STAT3, STAT5, and STAT6 to modulate symptom manifestation in allergy diseases [22]. Recent research work has also shown that CISH can regulate CCL26-mediated eosinophilic inflammation induced by interleukin-13 [23]. Hence, these findings suggest that CISH plays a key role in inflammation of many diseases [24].

SOCS family has become a cornerstone of intracellular signal transduction regulation since it was discovered in the 1990s. SOCS family involves eight members including cytokine-induced Src homology (SH2)-containing protein (CISH) and SOCS1-7. Cytokines, including interleukins (ILs), interferons (IFNs), and hematopoietic growth factors, activate the Janus kinase signal transducer and activator of transcription (JAK/STAT) pathway to elicit downstream effects in responding cells. According to their structural features, CISH is associated with cytokine signaling [25] acting as an important role in development, differentiation, and function of the immune response. 

The STAT protein family plays an important role in the JAK/STAT signaling pathway, but compared to the other isoforms, STAT3 is more conserved. According to previous studies, STAT3 is an important transcription factor for activation of multiple cytokines and growth factors, the activation of which can regulate genes responsible for proliferation, repression, migration, inflammation, and apoptosis depending on the cell type. Multiple cytokines are associated with the activation of STAT3 thereby regulating a variety of cellular functions. Hence, STAT3 has already attracted attention of researchers as an important therapeutic target in several diseases. A number of studies have also shown the importance of STAT3 in the inflammatory response to diseases as well as immune response such as inducing pro-inflammatory gene cyclooxygenase 2 expression to regulate TLR4-mediated inflammation in early and late phases, accelerating the development of colitis by the deficiency of STAT3 in hematopoietic cells leads, and involving in the inflammatory bowel disease and bacterial infections [25,26].

To sum up, the JAK/STAT pathway is one important member of intracellular signal transduction pathways. Studies have shown that the activation of JAK/STAT is crucial for inflammatory process, cellular apoptosis, and cell proliferation. CISH is one member of Suppressors of Cytokine Signaling (SOCS) family which acts as negative feedback suppressors of signal transduction and activator of transcription (STAT) family [24,27,28]. Besides inflammatory response, previous research also revealed STAT3, as an important transcription activator, encodes a series of genes to participate in the activities of downstream proteins such as apoptosis inhibitors and cell-cycle regulators [29,30]. Consequently, CISH/STAT3 regulation received our attention. On the basis of others’ previous research and our own mRNA-seq analysis, we furthermore proposed a reasonable hypothesis that electroacupuncture stimulation could enhance the repair process of injured muscle via CISH/STAT3 link to mediate activities of downstream factors. To verify this hypothesis, we detected these relevant indicators regarding Caspase3, BAX, Bcl-2, and CDKN2α through Western blotting and immunofluorescence (Figure 5).

In the early stage after injury, local necrotic muscle fibers stimulatingly secrete inflammatory molecules such as cytokines to facilitate inflammation [31]. These inflammatory cytokines are positively mediated by STAT family, especially STAT3 [32], and the absence of STAT3 represses inflammatory responses [25]. Recent work has shown that CISH can negatively regulate STAT proteins to attenuate inflammation, which plays an important role in the development of many diseases [21,22,23]. Notably, STAT3 resides in the cytoplasm and needs to be activated by protein phosphorylation to translocate into the nuclear thereby performing its transcriptional function [26]. In the current study, both expressions of CISH and STAT3 in J6O and J6N rats were down-regulated as compared to the rats from the NON group, and all of these results were with statistical differences. Both expressions of CISH and STAT3 in J6A rats were closer to NON rats. Furthermore, also noteworthy was higher level of CISH in the J6A group as compared to the J6O group (*p* < 0.05). Together with the result of electron microscopy, excessive jump training resulted in severe muscle damage and pronounced inflammatory infiltration possibly caused by the down-regulation of CISH to decrease STAT3 expression. Additionally, when compared to the natural recovery rats, rats with electroacupuncture stimulation showed more lessened infiltration of inflammatory cells and better structural recovery, suggesting the up-regulation of CISH may suppress the pro-inflammatory effects of STAT3 activation.

Regardless of the type of skeletal muscle injury, it is important to repress apoptosis for the general healing process, in which the Bcl-2 family associated with JAK/STAT activity determines whether the cell will die or live [33,34,35]. Presently, we found that rats with overuse injury from excessive jumps had an increased pro-apoptotic response as a result of their increased level of BAX relative to NON rats. Five days after jumping training, rats with electroacupuncture intervention demonstrated the significantly increased level of Bcl-2 and the decreased level of BAX, suggesting enhanced anti-apoptotic responses. Moreover, when compared to rats with natural recovery, electroacupuncture stimulation inhibited apoptosis better than natural healing as the result of the sharp up-regulation of Bcl-2 expression.

Cyclin-dependent kinase 2 (CDKN2α) gene, also known as multiple tumor suppressor l (MTS1), can produce the P16 protein, which negatively regulates the cell cycle and leads to cells in the prevention of G1/S transition by binding to CDK4 and CDK6 to inhibit cyclin D forming a kinase active complex with CDK4 [36]. This implies that decreased expression or deletion of CDKN2α could increase the ability of cell proliferation by lifting the inhibition of P16 against the cyclinD-CDK4 complex. Apoptosis occurs following induction of different stimuli. Besides the known responses of engulfing dying or dead cells, increasing studies have reported that apoptosis stimulates proliferation to promote tissue regeneration through metabolites released from apoptotic cells or a compensatory mechanism in which Caspase 3, the vital executioner in apoptosis, plays a major role [37,38]. Interestingly, when compared to NON rats, rats with jumping training up-regulated Caspase 3 and down-regulated CDKN2α. Five days after injury, when compared to NON rats, we found decreased Caspase 3 expression and slightly increased CDKN2α expression in natural healing rats, whereas increased Caspase 3 expression and decreased CDKN2α expression in rats with repeated electroacupuncture stimulation. These findings suggest that skeletal muscle fibers exposed to stressful conditions such as overused jumping could initiate the proliferation to promote morphological and functional recovery, and this proliferation would naturally diminish over time without any specific intervention following injury. In contrast, the electroacupuncture intervention after injury could keep the skeletal muscle in a good metabolic state, which is more conducive to the repair of damaged muscle.

Our study, however, has some limitations. As previously reported, muscle fibers are incapable of division because of their post-mitotic cell nature. Once injury happens, muscle fibers could not be repaired without the presence of satellite cells [31]. Meanwhile, numerous studies have supported that STAT3 is crucial for the myogenic progression of satellite cells following muscle injury [39]. Therefore, we will seek the relationship between CISH/ pSTAT3 regulation and satellite cells in the future research.

## 5. Conclusions

**Figure 8:**
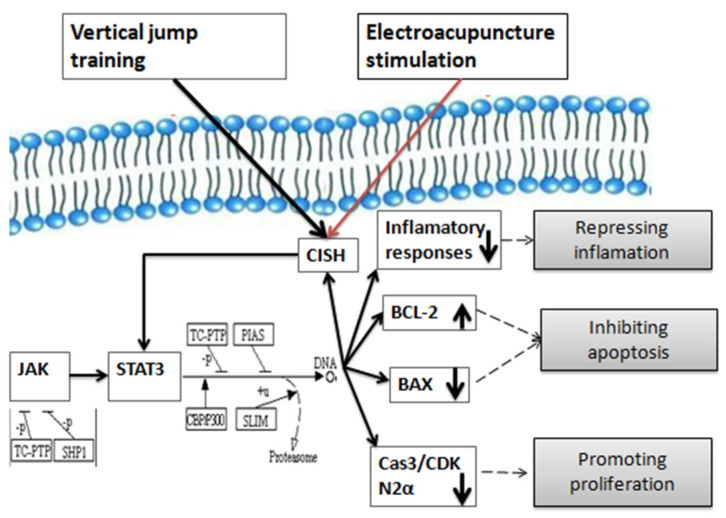
Schematic diagram of potential mechanism on the recovery of injured muscle underlying electroacupuncture stimulation at ST36.

We conclude that electroacupuncture stimulation at ST36 accelerates the recovery of the morphology and function of the damaged extensor digitorum longus via the up-regulation of CISH to modulate the activation of STAT3, thus suppressing the inflammatory responses, inhibiting apoptosis, and promoting proliferation of skeletal muscle (Figure 8).

## Figures and Tables

**Table 1:** Antibodies used for WB analysis of rats proteins.

Antibody Name	Molecular Weight (kDa)	Classification	Antibody Source	Catalog Number	Dilution Ratio
Caspase3	30	apoptosis	Servicebio	GB11009	1:1000
CDKN2α	17	cell cycle	Servicebio	GB13513	1:1000
STAT3	88	transcription factors	Servicebio	GB12176	1:1000
CISH	29	inflammatory	BOSTER	BA3758	1:1000
GAPDH	37	Internal reference	Servicebio	GB11002	1:1000

**Table 2:** Data matching and quality statistics.

Sample	Total Reads	Mapped Reads	GC(%)	Q20(%)	Q30(%)	Uniq Map	Multiple Map
NON1	46501974 (100%)	44400196 (95.48%)	49.7	98.09	94.58	41206966 (88.61%)	3193230 (6.87%)
NON2	49633854 (100%)	47481735 (95.66%)	50.4	98.05	94.4	43979527 (88.61%)	3502208 (7.06%)
NON3	43148380 (100%)	41288426 (95.69%)	50.01	98.31	95.02	38262260 (88.68%)	3026166 (7.01%)
J6O1	46373644 (100%)	44334550 (95.60%)	50.76	98.19	94.78	40978261 (88.37%)	3356289 (7.24%)
J6O2	51941888 (100%)	49654816 (95.60%)	50.48	98.23	94.81	45850954 (88.27%)	3803862 (7.32%)
J6O3	58064958 (100%)	55558156 (95.68%)	50.66	98.1	94.55	51541420 (88.77%)	4016736 (6.92%)
J6A1	40451452 (100%)	38442027 (95.03%)	49.4	98.22	94.95	35434601 (87.60%)	3007426 (7.43%)
J6A2	41286370 (100%)	39154025 (94.84%)	49.79	98.03	94.52	36119262 (87.48%)	3034763 (7.35%)
J6A3	44047722 (100%)	42032385 (95.42%)	49.5	98.3	95.01	38909299 (88.33%)	3123086 (7.09%)
J6N1	45749406 (100%)	43614333 (95.33%)	50.3	97.95	94.17	40441682 (88.40%)	3172651 (6.93%)
J6N2	59023458 (100%)	56409967 (95.57%)	50.55	98.09	94.54	52548642 (89.03%)	3861325 (6.54%)
J6N3	54380728 (100%)	52011006 (95.64%)	50.48	98.13	94.68	48213108 (88.66%)	3797898 (6.98%)

Notes: Total Reads: Number of Clean Reads, single-ended; Mapped Reads: Number Compared to reference genome and percentage of Clean Reads; GC (%): sample GC content; Q20(%): percentage of bases with mass value greater than or equal to 20; Q30(%): percentage of bases with mass value greater than or equal to 30; Uniq Mapped Reads: Number of Reads compared to unique position of reference genome and percentage of Clean Reads; Multiple Map Reads: compared number of Reads to Multiple locations in the reference genome and the percentage of Clean Reads.

**Table 3:** Co-expressed genes in KEGG and TOP GO pathways.

KEGG Signaling Pathway	Genes	Percentage	TOP GO Signaling Pathway	Annotated	Significant	Expected	KS
Endocytosis	6	8.57%	endocytosis	613	6	3.78	0.47256
Phagosome	8	11.43%	early phagosome	7	0	0.04	0.75058
Jak-STAT signaling pathway	5	7.14%	negative regulation of JAK-STAT cascade	50	1	0.31	0.0209
			JAK-STAT cascade	166	3	1.02	0.02164
			regulation of JAK-STAT cascade	147	3	0.91	0.07301
			positive regulation of JAK-STAT cascade	76	2	0.47	0.15578
			JAK-STAT cascade involved in growth hormone signaling pathway	7	0	0.04	0.18095
Calcium signaling pathway	5	7.14%	none				
Cell adhesion molecules CAMs	8	11.43%	none				
Ribosome	5	7.14%	regulation of ribosome biogenesis	4	0	0.02	0.09929
			rescue of stalled ribosome	2	0	0.01	0.26722
			negative regulation of ribosome biogenesis	2	0	0.01	0.31515
			ribosome disassembly	8	0	0.05	0.36302
			mature ribosome assembly	6	0	0.04	0.56498
			ribosome localization	17	0	0.1	0.62718
			establishment of ribosome localization	17	0	0.1	0.62718
			assembly of large subunit precursor of preribosome	4	0	0.02	0.71236
			ribosome assembly	113	2	0.7	0.78913
			ribosome biogenesis	417	6	2.57	0.91798
			90S preribosome assembly	11	0	0.07	0.99607
HTLV-I infection	10	14.29%	none				
PPAR signal pathway	6	8.57%	none				

## Data Availability

Not applicable.
